# Molecular typing, virulence traits and antimicrobial resistance of diabetic foot staphylococci

**DOI:** 10.1186/s12929-016-0250-7

**Published:** 2016-03-08

**Authors:** Carla Mottola, Teresa Semedo-Lemsaddek, João J. Mendes, José Melo-Cristino, Luís Tavares, Patrícia Cavaco-Silva, Manuela Oliveira

**Affiliations:** Centro de Investigação Interdisciplinar em Sanidade Animal, Faculdade de Medicina Veterinária, Avenida da Universidade Técnica, 1300-477 Lisbon, Portugal; BioFIG, Centro para a Biodiversidade, Genómica Integrativa e Funcional, Faculdade de Ciências, Universidade de Lisboa, Campo Grande, 1749-016 Lisbon, Portugal; Departamento de Medicina Interna, Hospital de Santa Marta/Centro Hospitalar de Lisboa Central, EPE, Rua de Santa Marta, 1169-024 Lisbon, Portugal; Faculdade de Medicina, Universidade de Lisboa, Instituto de Microbiologia, Avenida Prof. Egas Moniz, 1649-028 Lisbon, Portugal; TechnoPhage, S.A., Avenida Prof. Egas Moniz, 1600-190 Lisbon, Portugal; Centro de Investigação Interdisciplinar Egas Moniz (CiiEM), Instituto Superior de Ciências da Saúde Egas Moniz, Via Alternativa ao Monte de Caparica, 2829-511 Caparica, Portugal

**Keywords:** Diabetic foot staphylococci, PFGE, HA-MRSA, CA-MRSA, MLST, Virulence factors

## Abstract

**Background:**

Diabetes *mellitus* is a major chronic disease that continues to increase significantly. One of the most important and costly complications of diabetes are foot infections that may be colonized by pathogenic and antimicrobial resistant bacteria, harboring several virulence factors, that could impair its successful treatment. *Staphylococcus aureus* is one of the most prevalent isolate in diabetic foot infections, together with aerobes and anaerobes.

**Methods:**

In this study, conducted in the Lisbon area, staphylococci isolated (*n* = 53) from diabetic foot ulcers were identified, genotyped and screened for virulence and antimicrobial resistance traits. Genetic relationship amongst isolates was evaluated by pulsed-field-gel-electrophoresis with further multilocus sequence typing of the identified pulsotypes. PCR was applied for detection of 12 virulence genes and e-test technique was performed to determine minimal inhibitory concentration of ten antibiotics.

**Results:**

Among the 53 isolates included in this study, 41 *Staphylococcus aureus* were identified. Staphylococcal isolates were positive for intercellular adhesins *icaA* and *icaD*, negative for biofilm associated protein *bap* and pantone-valentine leucocidin *pvl. S. aureus* quorum sensing genes *agrI* and *agrII* were identified and only one isolate was positive for toxic shock syndrome toxin *tst*.

36 % of staphylococci tested were multiresistant and higher rates of resistance were obtained for ciprofloxacin and erythromycin. Clonality analysis revealed high genomic diversity and numerous *S. aureus* sequence types, both community- and hospital-acquired, belonging mostly to clonal complexes CC5 and C22, widely diffused in Portugal nowadays.

**Conclusions:**

This study shows that diabetic foot ulcer staphylococci are genomically diverse, present resistance to medically important antibiotics and harbour virulence determinants. These properties suggest staphylococci can contribute to persistence and severity of these infections, leading to treatment failure and to the possibility of transmitting these features to other microorganisms sharing the same niche. In this context, diabetic patients may become a transmission vehicle for microorganisms’ clones between community and clinical environments.

## Background

Foot ulcers are an increasing problem in patients with Diabetes *mellitus* and infection is a frequent complication that actually constitutes the most common cause of hospitalization in diabetic patients, often related to lower-extremity amputation [1]. Several studies have demonstrated that they represent an economic burden worldwide, comparable with the costs associated with cancer, depression, lung and musculoskeletal diseases [2, 3]. Diabetic foot infections (DFI) are often polymicrobial and can be caused by several pathogens, mainly Gram positive bacteria, being *Staphylococcus* the most predominant bacterial genus, as already described [4, 5].

*Staphylococcus* is a frequent commensal bacteria of human skin and mucosa, being one of the major cause of infections in humans, ranging from minor skin infections to severe infections such as septicaemia, endocarditis and osteomyelitis [6]. These bacteria may produce several virulence factors, one of the most important being biofilm formation, which consists in adherent bacterial populations growing inside their polymeric structures that confer the ability of evasion to immune system and to multiple antibiotic treatments [7]. Several virulence genes are implicated in biofilm formation, like *icaA* and *icaD*, responsible for the biosynthesis of polysaccharide intercellular adhesion (PIA) molecules, containing N-acetylglucosamine, the main constituent of the biofilm matrix in the accumulation phase [8]. A biofilm associated protein, coded by the gene *bap*, was also described as essential in biofilm production of some *Staphylococcus* spp. isolated from nosocomial infections [9].

One of the bacterial properties that allow the development and growth of multicellular biofilm is cell communication and signalling, in which the bacterial signals reach a specific density or “quorum” activating regulatory genes that control some cellular processes [10]; the *S. aureus* accessory gene regulator (*agr*) was the first peptide signal discovered [11].

Many virulence determinants including toxins, tissue degrading enzymes and immune evasion factors, are secreted by staphylococci, particularly by *S. aureus* [12]. *Clfa* is a gene responsible for causing platelet activation through binding to fibrinogen and fibrin and for inhibiting phagocytosis in *S. aureus* [13]. One of the major threats in severe tissue necrosis is the presence of the cytotoxin panton-valentine leukocidin (*pvl*), whose locus is carried on a bacteriophage, manifesting commonly in strains isolated from community-acquired skin and soft tissue infections and especially from pneumonia [14]. Some *S. aureus* isolates also secrete the toxic shock syndrome toxin 1 (TSST-1), a superantigenic toxin responsible for staphylococcal scarlet fever and toxic shock syndrome, encoded by the *tst* gene [15]. *S. aureus* and coagulase-negative staphylococci (CoNS) infections occur in the community or in healthcare settings and an extremely high percentage of these isolates are resistant to methicillin. In Europe, methicillin-resistant *S. aureus* (MRSA) are predominantly acquired in healthcare settings representing a major challenge to the control of antibiotic resistance in hospitals [16]. Portugal is one of the European countries presenting higher rates of MRSA in hospitals, reaching 53.8 % according to last report data [17], and hospital-associated MRSA (HA-MRSA) have been extensively characterized [18–20]. However, less is known about the epidemiology of MRSA in the community (CA-MRSA), which remains poorly understood [21]. Epidemic MRSA (EMRSA)-15 clone (ST22-IV), is currently the most predominant clone in Portuguese hospitals, accounting for 72 % of all MRSA isolates, followed by the NY/Japan clone (NY/JP) (ST5-II). More recently a variant of this clone (ST105) appeared as the second most predominant clone in Portuguese hospitals [20, 22].

In the last years the complications of DFI have raised due to the increased rate of multidrug-resistant (MDR) isolates, so a better knowledge of these bacteria is necessary in order to institute an effective antibiotic therapy [1, 5]. This study aimed to investigate the molecular types, virulence traits and antimicrobial susceptibility pattern of *Staphylococcus* spp. isolated from diabetic foot ulcers in Portugal.

## Methods

### Bacterial isolates

A total of 53 staphylococci clinical isolates from diabetic foot ulcers, belonging to 49 samples collected in a transversal observational study conducted at four clinical centers in Lisbon, from January 2010 to July 2010 [4], were used in this study. Only eight patients were hospitalized during the collection of samples. All isolates were processed, isolated and identified by standard methods [4]. Each isolate corresponds to a different patient, with the exception of following pairs, which belonged to the same patient: *S. aureus* A2-1a and A2-1b, *S. aureus* B3-2 and B3-3, *S. aureus* Z1-1 and Z1-2, *S. aureus* Z3-1 and Z3-2, *S. aureus* Z21-1 and Z-21-3, *S. aureus* Z27-2 and Z27-3 and *S. aureus* Z33-1 and Z33-2. Although being recovered from the same patient, such staphylococci were included in further analysis due to the distinct colony morphologies observed during isolation and purification procedures.

### Identification at species level

After inoculation in Columbia Agar + 5 % sheep blood (Biomerieux), plates were incubated at 37 °C for 24 h. Rapid DNA extraction was performed by suspending four to five bacterial colonies in 100 μL of TE (10 mM Tris, 1 mM EDTA, pH 7.8) buffer and heating to 97 °C for seven min. After centrifugation at 15 000 g for five min, supernatant was collected and stored at −20 °C for subsequent PCR screening.

Staphylococcus *aureus* and *Staphylococcus epidermidis* identification was confirmed using a multiplex PCR protocol described elsewhere [23]. Amplified products were analysed by electrophoresis using 0.5X Tris-Borate-EDTA (TBE) buffer in a 2 % agarose gel (Bioline) stained with GreenSafe (NZYTech) and visualized by transillumination under UV (Pharmacia Biotech, Thermal Imaging System FTI-500). NZYDNA ladder VI (NZYTech) was used as a molecular weight marker. *S. aureus* ATCC 29213 and *S. epidermidis* ATCC 35984 were used as PCR amplification controls.

For the remaining staphylococcal isolates, Biomerieux API Staph galleries were used for species identification.

### Screening for virulence factors

The presence of virulence determinants was evaluated by PCR amplification using primers and protocols previously described. Genes tested included coagulase gene *coa* [24], protein A gene *spa* [24], adhesin genes *icaA* and *icaD* [25], biofilm associated protein gene *bap*, clumping factor a *clfa* [24], accessory regulators genes *agrI*, *agrII*, *agrIII* and *agrIV* [26], toxic shock syndrome toxin 1 gene *tst* and panton-valentine leukocidin *pvl* [27].

*S. aureus* ATCC 25923 was used as an amplification control for *coa*, *spa* and *clfa* genes. *S. epidermidis* ATCC 35984 was used as *icaA* and *icaD* positive control*. S. aureus bap* positive control was kindly provided by Dr. Penadés (Cardenal Herrera University, Valencia, Spain), *agrI*, *agrII*, *agrII* e *agrIV* control strains by Dr. Carmen Torres (Rioja University, Spain), and *tst* and *pvl* positive controls by Dr. Michèle Bes (Centre National de Reference des Staphylocoques, Lyon,Frande).

### Evaluation of antibiotic susceptibility and detection of *mecA*

Minimal inhibitory concentrations (MIC) were determined for antibiotics: cefoxitin (Fox), ceftaroline (Cpt), ciprofloxacin (Cip), clindamycin (Cli), doxycycline (Dox), erythromycin (Ery), gentamicin (Gen), linezolid (Lzd), meropenem (Mem) and vancomycin (Van), by placing e-test strips (Biomérieux) on staphylococci inoculated on Mueller Hinton plates, incubated for 24 h at 37 °C. Test performance was monitored using *S. aureus* ATCC 29213.

Detection of *mecA* gene was performed as previously described [23]. Amplified products were analysed by electrophoresis with 0.5X Tris-Borate-EDTA (TBE) buffer in a 1.5 % agarose gel (Bioline) stained with GreenSafe (NZYTech) and visualized by transillumination under UV (Pharmacia Biotech, Thermal Imaging System FTI-500). NZYDNA ladder VI (NZYTech) was used as molecular weight marker. MRSA control strain was kindly provided by Dr. Birgit Strommenger (Robert Koch Institute, Germany).

Staphylococci under analysis were defined as Methicillin Resistant Staphylococcus (MRS) if resistant by cefoxitin MIC or if *mecA* positive [28], and as Multi-drug Resistant (MDR) if resistant to three or more antimicrobials belonging to different antibiotic classes and bacterial targets [29].

### Macrorestriction analysis by Pulsed-Field Gel Electrophoresis (PFGE)

Molecular fingerprinting of staphylococci was performed by PFGE using a CHEF-DRIII apparatus (Bio-Rad Laboratories, San Diego, USA). Bacterial cultures were grown overnight on Columbia agar supplemented with 5 % sheep blood (BioMérieux) and a cellular suspension of 5 × 10^9^ CFU/mL incorporated into 1.5 % low melting point agarose (BioRad). Discs were immersed into a lysis solution with lysostaphin (Sigma-Aldrich) (50 μg/ml), lysozyme (Merck) (100 μg/ml) and RNase (Roche) (50 μg/ml) at 37 °C for 3 h. After lysis, discs were incubated with proteinase K (NZYTech, Portugal) (1 mg/ml) for 17 h at 50 °C, followed by overnight digestion with *Sma*I (Takara) at 25 °C. Digested DNA was submitted to electrophoresis in 1 % agarose gel (Seakem LE) for 23 h at 14 °C and 6 V/cm with pulse times of five to 35 s. Lambda Ladder PFG Marker (BioLabs) 50 μg/ml was used as molecular weight marker. Agarose gels were stained with ethidium bromide and visualized by transillumination under UV (Pharmacia Biotech, Thermal Imaging System FTI- 500). BioNumerics 7.5 software (Applied Maths, Kortrijk, Belgium) was used to register macrorestriction patterns and clustering analysis was performed using DICE similarity coefficient and the unweighted-pair group method with arithmetic mean (UPGMA).

### *S. aureus* multilocus sequence typing (MLST)

Amplification of seven housekeeping genes, including carbamate kinase *arcC*, shikimate dehydrogenase *aroE*, glycerol kinase *glpF*, guanylate kinase *gmk*, phosphate acetyltransferase *pta*, triosephosphate isomerase *tpi*, and acetyl coenzyme A acetyltransferase *yqiL*, was done according to the already published protocols [30]. DNA sequencing was performed by Stabvida (Portugal). MLST sequences were analysed using Bionumerics 7.5 software (Applied Maths, Kortrijk, Belgium) and sequence types (ST) assigned according to the *S. aureus* MLST database (http://saureus.mlst.net) The eBURST algorithm, available at (http://eburst.mlst.net), was used to classify different ST into clusters or clonal complexes (CC). A minimum spanning tree (MST) constructed with BioNumerics 7.5 software (Applied Maths, Kortrijk, Belgium) using the concatenated seven gene fragments was also used to evaluate the phylogenetic relationships between isolates.

## Results

### Identification at species level

Among the 53 isolates included in the study, 41 were identified as *S.aureus* and six as *S. epidermidis* by multiplex PCR. The API galleries identified two isolates as *S. haemolyticus*, one as *S. schleiferi*, one as *S. caprae*, one as *S. simulans* and one as *Staphylococcus* sp.

### Screening for virulence factors

**Fig 1:**
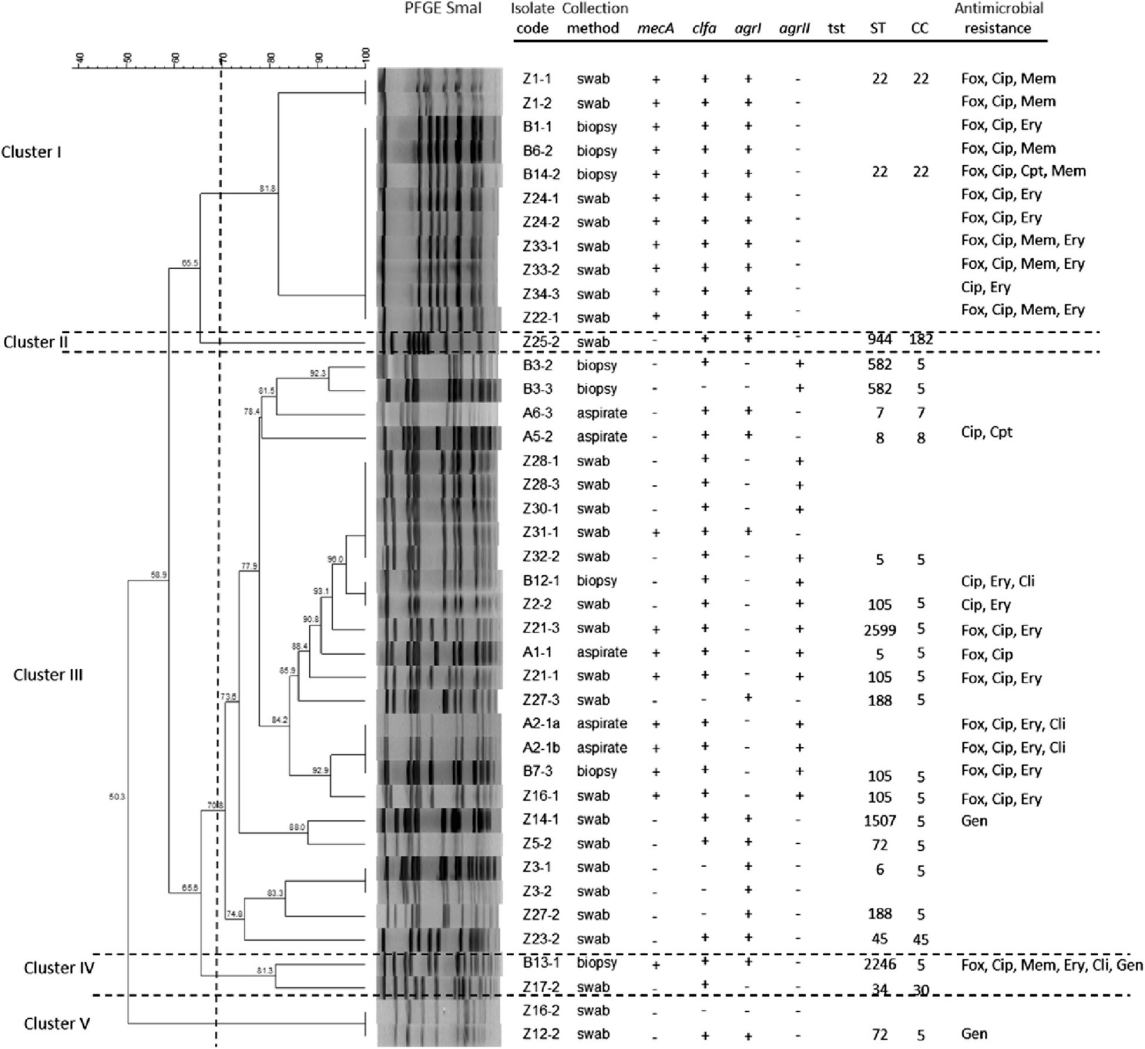
Dendrogram based on *Sma*I-PFGE patterns of the *S. aureus* diabetic foot isolates. The image also displays information regarding sample collection method, presence of virulence genes, ST/CC allocation and antimicrobial resistance profile. Fox - cefoxitin; Cip – ciprofloxacin; Mem – meropenem; Ery – erythromycin; Cpt – ceftaroline; Cli – clindamycin; Gen – gentamicin

All isolates were positive for *icaA* and *icaD* and negative for *bap* and *pvl*. The *clfa* gene was present in 70 % of the isolates (*S. aureus n* = 30, *S. epidermidis n* = 3 and *S.* sp *n* = 1). The *S. aureus quorum sensing* genes *agrI* and *agrII* were present in 60 % and 40 % of the *S. aureus* isolates respectively, and no *agrII* or *agrIV* were found. Two *S. aureus* isolates did not harbour *agr*. With the exception of two isolates (one of which also *agr* negative), all *S. aureus* were positive for *spa*. As expected, all *S. aureus* isolates were *coa* positive*,* whilst only one *S. aureus* was positive for *tst* and it was MSSA (Fig. 1).

### Evaluation of antibiotic susceptibility and detection of *mecA*

All isolates were considered susceptible to vancomycin (MIC ≤ 2 μg/mL) and presented the same susceptibility to linezolid (MIC ≤ 4 μg/mL) and doxycycline (MIC ≤ 4 μg/mL) with the exception of one methicillin-resistant *S. epidermidis* (MRSE) isolate (MIC ≥ 8 μg/mL and MIC ≥ 16 μg/mL for linezolid and doxycycline respectively), which was resistant to six of the antibiotics tested. Ceftaroline MIC values were ≤ 0.5 μg/mL and only two MRSA presented MIC ≥ 4 μg/mL (ceftaroline-resistant). About 90 % of isolates obtained MIC values for clindamycin of ≤ 0.25 μg/ml and for gentamicin 4 ≤ μg/ml. About 57 % of isolates were considered susceptible to ciprofloxacin (≤4 μg/ml) and eythromycin (≤8 μg/ml), presenting a resistance rate of 43 %. The percentage of MDR isolates was 36 % (Fig. 1).

Among the 41 *S. aureus* isolates tested, 20 were classified as MRSA (*mecA* positive) (Fig. 1), resulting in a prevalence of 48.7 % among *S. aureus* carriers; of these, 14 were cefoxitin resistant. Among the six *S. epidermidi*s isolates, five were MRSE (*mecA* positive) and 3 were cefoxitin resistant. The other *Staphylococcus* isolates didn’t carry the *mecA* gene and were cefoxitin susceptible. The total prevalence of methicillin-resistant isolates was 47 %.

### Macrorestriction analysis by Pulsed-Field Gel Electrophoresis -PFGE-

Analysis of the dendrogram displayed in Fig. 1 led to the selection of a 70 % similarity level for the assignment of PFGE genomic types (pulsotypes). Hence, *Sma*I-macrorestrition analysis revealed 18 distinct genomic patterns among the 41 *S. aureus* isolates examined. Cluster analysis allowed grouping the isolates into five main clusters at approximately 70 % similarity with one single member cluster (Fig. 1). All isolates included in cluster I were MRSA, *clfa* and *agrI* positives and belonged to ST22 (CC22). They were all resistant to ciprofloxacin and most of them also to erythromycin. Cluster II included only one isolate, sensitive to all antibiotics tested, *clfa* and *agrI* positive and belonging to ST944 (CC182). The *agrII* positive isolates were located only in cluster III that was the more diverse group because included different genoypes, most of them *clfa* positive belonging to CC5, both MRSA and MSSA. These MRSA isolates showed resistance to ciprofloxacin and erythromycin. The only one MSSA *agrII* isolate that was *tst* positive, belonged to this group. Cluster IV included two different genotypes, one of which stood out (B13-1), being resistant to six of the antibiotics tested. Cluster V included two MSSA isolates, one *clfa*-*agrI* positive and the other *clfa*-*agr* negative. Regarding the six *S. epidermidis* isolates, although the number was inferior, four pulsotypes were observed and, noteworthy, two different pulsotypes corresponded to two isolates obtained from the same patient (data not shown).

### *S. aureus* multilocus sequence typing (MLST)

**Fig 2:**
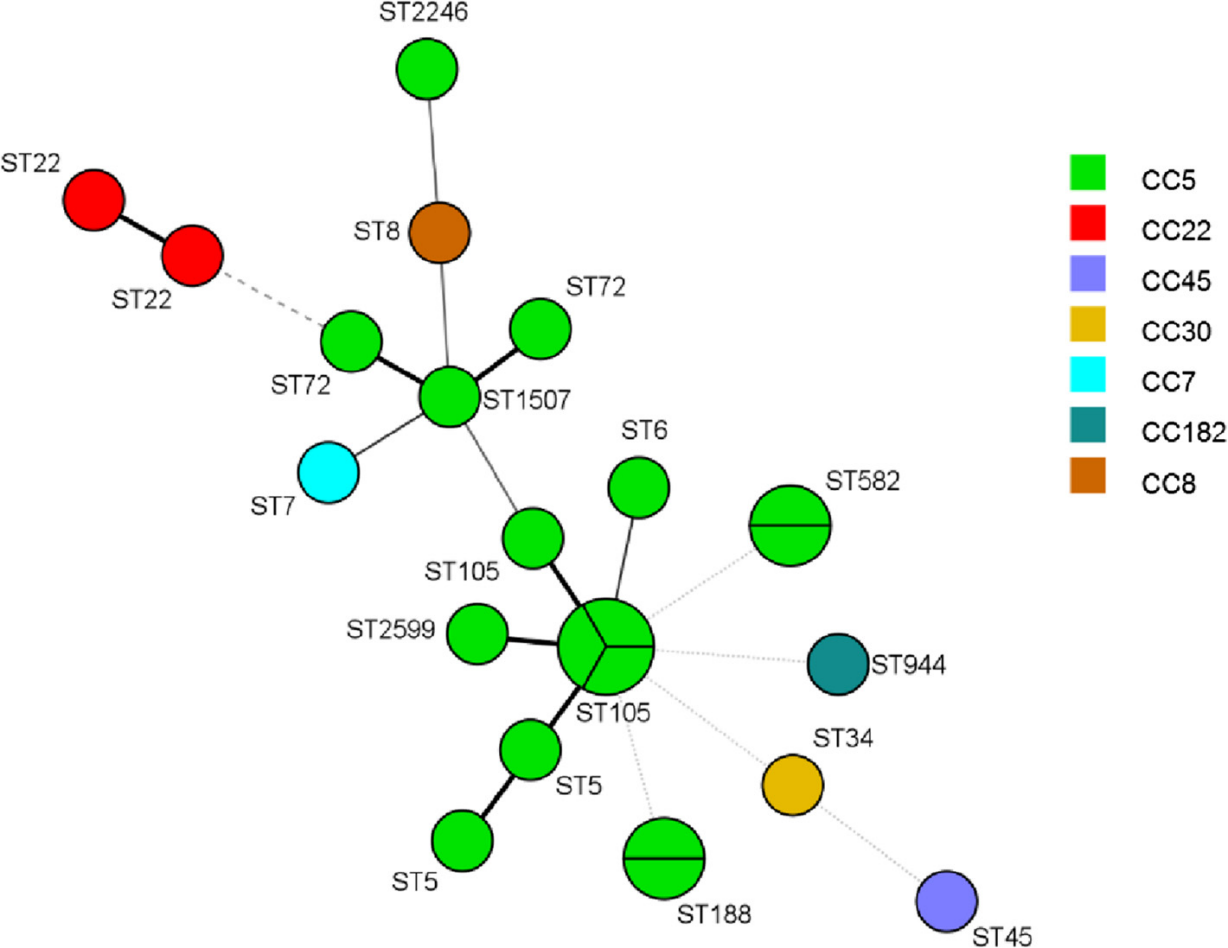
Minimum spanning tree of 23 *S. aureus* representing the 23 different pulsotypes detected amongst the diabetic foot isolates. Nodes indicate sequence type (ST) and their size shows the relative number of isolates for each ST. Every colour represents a distinct clonal complex

High genetic diversity was revealed by MLST, as indicated by the detection of 15 ST among the 23 isolates tested (Fig. 1). Briefly, ST105 (*n* = 4), ST5 (*n* = 2), ST22 (*n* = 2), ST188 (*n* = 2 in the same patient with two different pulsotypes), ST582 (*n* = 2 in the same patient with two different pulsotypes), ST6 (*n* = 1), ST7 (*n* = 1), ST8 (*n* = 1), ST34 (*n* = 1), ST45 (*n* = 1), ST 72 (*n* = 1), ST944 (*n* = 1), ST1507 (*n* = 1), ST2246 (*n* = 1), ST2599 (*n* = 1, in a patient with a ST105 also) (Fig. 1 and Fig. 2). Based on sequence typing, isolates were assigned to seven MLST CC: CC5 (*n* = 17, including the two different pulsotypes found in two patients), CC22 (*n* = 2), CC7 (*n* = 1), CC8 (*n* = 1), CC30 (*n* = 1), CC45 (*n* = 1), CC182 (*n* = 1) (Fig. 1). MRSA lineages included ST105 (CC5), ST5 (CC5), ST22 (CC22) and ST2599 (CC5). The only MSSA *tst* positive isolate belonged to ST5. The minimum spanning tree (MST) shows the phylogenetic relationships among diabetic foot staphylococci (Fig. 2).

## Discussion

Although previous studies reported *Pseudomonas aeruginosa* as the most common isolate in DFU [31, 32], many others authors from the late 1990s have shown that Gram positive cocci are the most predominant agents responsible for DFI, with *S. aureus* being the most commonly isolated pathogen with considerably high rates of MRSA [33, 34]. According to our results, most isolates were identified as *S. aureus* (77.3 %) and 48.7 % of them considered MRSA. A study conducted by Sotto et al. [35] reported a similar MRSA percentage but several studies showed lower rates [5, 32, 36]. The highest MRSA percentages in DFI, reaching 70 %, were found in India [37].

Regarding antimicrobial susceptibility patterns, 34 % of all staphylococcal isolates were cefoxitin resistant. However, *mecA* detection is considered the gold standard for methicillin-resistance by the CLSI [28] and 47 % of the studied isolates were *mecA* positive.

Only 10 % of all staphylococci showed resistance to meropenem but MRS isolates should be considered resistant to other β-lactam agents, therefore including also meropenem, because most cases of documented MRS infections have responded poorly to β-lactam therapy [28]. Cephalosporins with anti-MRSA activity include ceftaroline, the active metabolite of ceftaroline fosamil (Teflaro®, Forest Laboratories), a cephalosporin with an *in vitro* broad spectrum against MRSA and most enteric organisms [38]. Ceftaroline, approved by U.S. Food and Drug Administration (FDA) for treatment of acute bacterial skin infections, displayed a very good efficacy in the studied isolates with MIC value ≤0.5 μg/mL, with the exception of two resistant MRSA isolates. It is important to refer that one MRSA isolate that was resistant to six antibiotics studied showed sensitivity to ceftaroline. These results are in agreement with some recent studies that have already shown the excellent activity of ceftaroline, both in vitro [39] and in vivo [40].

Resistance to linezolid and doxycycline was detected only in one MRSE that showed resistance to six antibiotics. Linezolid-resistance in *S. epidermidis* has been already reported [41], possibly linked to a mutation in the V region of the 23S rRNA gene. MIC values for clindamycin and gentamicin showed susceptibility for 90 % of the isolates. These results suggest a good efficacy of linezolid, doxycycline, clindamycin and gentamicin for DFI treatment [42, 43]. As expected, all *Staphylococcus* tested were susceptible to vancomycin; until today only one case of vancomycin-resistant *S. aureus* was described in Europe, Portugal [44], and few cases worldwide, mostly in the USA [45].

About 43 % of the isolates were resistant to ciprofloxacin and erythromycin, two antibiotics largely used in clinical practice for these type of infections. Similar rates in ciprofloxacin and erythromycin resistance were found in a study conducted by Gadepalli et al. [36]. With the increasing use of quinolones in clinical practice, the development of resistance mutants has increased [46], pointing out for the importance to their careful administration in clinical settings. Several genes are implicated in macrolide resistance, especially in staphylococci and streptococci [47], explaining the low susceptibility rates of erythromycin in this bacterial genus.

It’s important to remember that DFI are generally polymicrobial and the choice of antibiotic therapy often doesn’t target specific pathogens. In fact, the present investigation revealed a high rate (36 %) of MDR isolates in DFI, which is in accordance with other reports [33, 36, 48] and should represent a serious warning for the control of this type of infections.

Virulence factors, like surface proteins and extracellular toxins, are widely distributed among staphylococci, potentially causing harmful pathogenic effects to the host [14]. In this study two *S. aureus* isolates were *spa* negative. Some studies have already reported the absence of *spa* protein with percentages of 3–5 % [49], that seems to be linked to point mutations. In a recent study we demonstrated that the staphylococcal isolates are able to form biofilm [50] which may explain why all isolates tested were positive for *icaA* and *icaD*. Otherwise, none of the isolates carried the *bap* gene, already described in some *Staphylococcus* spp. isolated from nosocomial infections [9].

The *clfA* gene was present in 70 % of our isolates including some *S. epidermidis*. The presence of *clfa* in *S. epidermidis* can be justified by the fact that in this species the fibrinogen-binding proteins SdrG or Fbe, associated to adherence to fibrinogen, are highly similar to *S. aureus* clumping factors A and B [51].

The only *tst*-positive isolate was a MSSA, *agrII*-positive belonging to ST5. Jarraud et al. in [27] reported that most *tst*-positive *S. aureus* strains were associated with both community and hospital-acquired diseases and were all methicillin-sensitive *S. aureus* (MSSA).

None of our isolates was *pvl*-positive. The *pvl* locus is strongly associated to CA-MRSA and often to *agr* group *III* background [52]. In Portugal, it was related with one case of CA-MRSA in 2012, belonging to the USA300 epidemic clone (ST8-IVa, t008, *pvl* positive), the predominant CA-MRSA clone in USA at present [53]. The USA300 is a rare clone in Europe and its low prevalence in Portugal was expected [56].

The contribution of the *agr* system to *S. aureus* virulence by gene regulation has already been described, as well as the association of a particular *agr* type in clinical isolates harbouring important virulence factors [54]. The *agr* group *I* was identified in the majority of the analysed staphylococci, followed by *agrII*, as previously reported in numerous other studies [27, 55]. Neither *agrIII* nor *agrIV* were observed. Two *S. aureus* isolates were *agr*-negative, but it is known that these variants can occur both in vivo and in vitro [56].

The virulence profile of the studied DFI isolates was more similar to CA-MRS than HA-MRS strains. This is an unusual finding, considering that diabetic patients attend frequently healthcare facilities and may suggest an increasing lack of barrier between both settings: hospital and community.

Sotto et al. in [35] demonstrated that the virulence gene profiles of DFI *S. aureus* isolates enables to distinguish the grade of ulcers and to predict its outcome; more knowledge about the virulence features of DFI isolates would be very helpful in establishing a more accurate diagnosis and consequently an adequate therapy.

PFGE genomic typing demonstrated a high diversity of clones, detecting 18 *S. aureus* and four *S. epidermidis* pulsotypes, respectively. According to Tenover et al. 1995 [57], it is highly probable that *S. aureus* isolates grouping in the same pulsotype with 100 % similarity belong to the same ST, as determined by MLST. The correlation between PFGE and MLST showed that PFGE cluster I was the most homogeneous cluster, including only MRSA ST22 (CC22) isolates, the most common ST observed in this study. Portugal is the European country with the highest rate of MRSA (54.6 %) [3] and CC22 is a common and widespread clonal group from which different MRSA have emerged, like the pandemic ST22-MRSA-IV (UK-EMRSA 15), present in hospitals as well as in outpatients [58]. CC22 represents a major clone in Portugal hospitals since 2001, having replace the Brazilian clone [59], and its prevalence has increased to more than 70 % of MRSA, likewise to what is observed in the United Kingdom, where this clone is believed to have originated [22]. All ST22 isolates were positive for *clfa*, another virulent factor that confers pathogenicity, and presented the quorum sensing *agr I* gene, already described as being common in ST22 staphylococci [59, 60].

The most common CC isolated in our study was CC5, present in PFGE clusters III, IV and V, and ST5 represented the second most frequent ST, after ST22. CC5 is another common and widespread clonal complex that includes a large number of different MRSA, some of which pandemic [58]. Shortly after the emergence of EMRSA-15, the New York-Japan (NY/JP) ST5-II and, more recently, a variant of this clone, ST105-II, appeared as the second most predominant clone in Portuguese hospitals [20]. Recently, a high percentage of MRSA (21.6 %) was also found in a community in Portugal, where EMRSA-15 or related clones were the predominant ones (77.2 %), followed by NY/JP or related clones (14.9 %) [61]. In this study, isolates belonging to CC5 presented mainly *agr* type *II*, particularly ST5, and included both MRSA and MSSA [18].

Besides ST5 and ST105, several ST belonging to CC5 were identified, namely MSSA *agrI* ST6, MSSA *agrI* ST72, MSSA *agrI* ST188, MSSA *agrI* ST582, MSSA *agrI* ST1507, MRSA *agrI* ST2246 and MRSA *agrII* ST2599. These less frequent ST have already been described in Portugal [20, 61, 62], with the exception of the ST1507 and ST2599, but little information is available regarding these ST. In fact, the only description found in the *S. aureus* MLST database (http://saureus.mlst.net), refers to a MRSA ST1507 isolated in 2006 in South Korea from a foodborne source and a MRSA ST2599 isolated from urine in 2013 in the USA. In our study the patient from which ST2599 (CC5) was recovered, also presented another *S. aureus* belonging to ST105 (CC5), being the only case where it was possible to identify two different ST in the same patient. In the other six cases in which the same patient showed two similar, but not identical pulsotypes, MLST revealed that they belonged to the same ST. Interestingly, some clones belonging to different CC presented a higher PFGE similarity than clones included in the same CC, as already observed [18].

Cluster II included only one isolate, MSSA *agrI* ST944 (CC182). MSSA ST944 was described in Switzerland being isolated from nasal swabs of healthy risk-free adult carriers [63] and in China, where it was present with high frequency in nasal carriage of healthy children in a kindergarten [64]. In the *S. aureus* MLST database, a MSSA ST944 has also been described in Norway, related with nasal swab carriage (http://saureus.mlst.net).

Cluster III was the most heterogeneous cluster, including mainly MSSA *agrI* isolates, belonging to the following ST: ST7 (CC7), ST8 (CC8), ST34 (CC30) and ST45 (CC45). In fact, a previous study concerning the population structure of MSSA in Portugal showed that these CC were, among others, the most predominant clonal types found between 1992 and 2011, both in the community and hospitals settings [20].

Patients with DFI constantly attend clinical centres for wounds healthcare, which may explain the high diversity of pulsotypes and ST found, including the main hospital-acquired clones present in Portugal (CC5 and CC22). It is important to refer that several less frequent clones, seldom described in literature and MLST database, were also found in this study. Therefore, diabetic patients can be important vehicles for clonal dissemination from the hospitals into the community and contrariwise, including less common clones.

## Conclusions

To our knowledge this is one of the few reports of staphylococci isolated from DFI that include information about the isolates origin, virulence factors and antimicrobial resistance profiles. Studies in DFI microbiology are scarce, as described recently by Zenelaj et al. [5], and further investigation of diabetic foot infections is urgent, allowing to adapt the therapeutic approach of these patients to the microbiological characteristics of the microorganisms involved.

## Abbreviations

*agr*: acessory gene regulator
CA-MRSA: community-adquired MRSA
CC: clonal complex
CIP: ciprofloxacin
CLI: clindamycin
CoNS: coagulase-negative staphylococci
CPT: ceftaroline
DFI: diabetic foot infections
DFU: diabetic foot ulcers
DOX: doxycycline
EMRSA: epidemic MRSA
ERY: erythromycin
FOX: cefoxitin
GEN: gentamicin
HA-MRSA: hospital-associated MRSA
ica: intercellular adhesin
LZD: linezolid
MDR: multidrug-resistant
MEM: meropenem
MIC: minimum inhibitory concentration
MLST: multilocus sequence typing
MRS: methicillin- resistant *Staphylococcus*
MRSA: methicillin-resistant *S. aureus*
MRSE: methicillin-resistant *S. epidermidis*
MST: minimum spanning tree
PFGE: pulsed-field gel electrophoresis
*pvl*: panton-valentine leukocidin
ST: sequence type
*S. aureus*: *Staphylococcus aureus*
*S. epidermidis*: *Staphylococcus epidermidis*
VAN: vancomycin

## References

[1] Spichler A, Hurwitz BL, Armstrong DG, Lipsky BA (2015). Microbiology of diabetic foot infections: from Louis Pasteur to “crime scene investigation.”. BMC Med.

[2] Rice JB, Desai U, Cummings AKG, Birnbaum HG, Skornicki M, Parsons NB (2014). Burden of diabetic foot ulcers for medicare and private insurers. Diabetes Care.

[3] Van Acker K, Léger P, Hartemann A, Chawla A, Kashif Siddiqui M: Burden of diabetic foot disorders, guidelines for management and disparities in implementation in Europe: a systematic literature review. Diabetes Metab Res Rev 2014, 30:635–645. 10.1002/dmrr.2523 24470359

[4] Mendes JJ, Marques-Costa A, Vilela C, Neves J, Candeias N, Cavaco-Silva P, Melo-Cristino J. Clinical and bacteriological survey of diabetic foot infections in Lisbon. Diabetes Res Clin Pract. 2012;95:153–61. 10.1016/j.diabres.2011.10.001 22019426

[5] Zenelaj B, Bouvet C, Lipsky BA, Uckay I (2014). Do Diabetic Foot Infections With Methicillin-Resistant Staphylococcus aureus Differ From Those With Other Pathogens?. Int J Low Extrem Wounds.

[6] Harastani HH, Araj GF, Tokajian ST (2014). Molecular characteristics of Staphylococcus aureus isolated from a major hospital in Lebanon. Int J Infect Dis.

[7] Sekhar S, Ohri M, Chakraborti A, Mendez-Vilas A (2010). Biofilms: an evolving and universal evasive strategy of bacterial pathogens. Current Research, Technology and Education Topics in Applied Microbiology and Microbial BioTechnology.

[8] Cos P, Tote K (2010). Biofilms: an extra hurdle for effective antimicrobial therapy. Curr Pharm Des.

[9] Potter A, Ceotto H, Giambiagi-Demarval M, dos Santos KRN, Nes IF, Bastos MDCDF (2009). The gene bap, involved in biofilm production, is present in Staphylococcus spp. strains from nosocomial infections. J Microbiol.

[10] Sifri CD (2008). Healthcare epidemiology: quorum sensing: bacteria talk sense. Clin Infect Dis.

[11] Novick RP, Geisinger E (2008). Quorum sensing in staphylococci. Annu Rev Genet.

[12] Gordon RJ, Lowy FD (2008). Pathogenesis of methicillin-resistant Staphylococcus aureus infection. Clin Infect Dis.

[13] Chambers HF, Deleo FR (2010). Waves of Resistance: Staphylococcus aureus in the Antibiotic Era. Nat Rev Microbiol.

[14] Holmes A, Ganner M, McGuane S, Pitt TL, Cookson BD, Kearns AM (2005). Staphylococcus aureus isolates carrying Panton-Valentine leucocidin genes in England and Wales: frequency, characterization, and association with clinical disease. J Clin Microbiol.

[15] Durand G, Bes M, Meugnier H (2006). New methicillin-resistant Staphylococcus aureus clones containing the toxic shock syndrome toxin 1 gene responsible for hospital-and community-acquired infections. J Clin Microbiol.

[16] Grundmann H, Schouls LM, Aanensen DM, Pluister GN, Tami A, Chlebowicz M, Glasner C, Sabat AJ, K W, O H, Friedrich AW: The dynamic changes of dominant clones of Staphylococcus aureus causing bloodstream infections in the European region : Results of a second structured survey. Euro Surveill 2014, 19(Suppl 49):1–10. 10.2807/1560-7917.es2014.19.49.20987 25523972

[17] European Centre for Disease Prevention and Control (ECDC): Antimicrobial Resistance Surveillance in Europe 2012. 2012.

[18] Aires de Sousa M, Conceicão T, Simas C, De Lencastre H (2005). Comparison of Genetic Backgrounds of Methicillin-Resistant and -Susceptible Staphylococcus aureus Isolates from Portuguese Hospitals and the Community. J Clin Microbiol.

[19] Amorim ML, Vasconcelos C, Oliveira DC, Azevedo A, Calado E, Faria NA, Pereira M, Castro AP, Moreira A, Aires E, Cabeda JM, Ramos MH, Amorim JM, de Lencastre H. Epidemiology of methicillin-resistant Staphylococcus aureus (MRSA) nasal colonization among patients and healthcare workers in a Portuguese hospital: a pre-intervention study toward the control of MRSA. Microb Drug Resist. 2009;15:19–26. 10.1089/mdr.2009.0881 19296773

[20] Tavares A, Faria NA, De Lencastre H, Miragaia M (2014). Population structure of methicillin-susceptible Staphylococcus aureus (MSSA) in Portugal over a 19-year period (1992–2011). Eur J Clin Microbiol Infect Dis.

[21] Almeida ST, Nunes S, Paulo ACS, Faria NA, de Lencastre H, Sá-Leão R (2014). Prevalence, risk factors, and epidemiology of methicillin-resistant Staphylococcus aureus carried by adults over 60 years of age. Eur J Clin Microbiol Infect Dis.

[22] Faria NA, Miragaia M, Lencastre, The Multi Laborat H (2013). Massive Dissemination of Methicillin Resistant Staphylococcus aureus in Bloodstream Infections in a High MRSA Prevalence Country: Establishment and Diversification of EMRSA-15. Microb Drug Resist.

[23] Pereira EM, Schuenck RP, Malvar KL, Iorio NLP, Matos PDM, Olendzki AN, Oelemann WMR, dos Santos KRN. Staphylococcus aureus, Staphylococcus epidermidis and Staphylococcus haemolyticus: methicillin-resistant isolates are detected directly in blood cultures by multiplex PCR. Microbiol Res. 2010;165:243–9. 10.1016/j.micres.2009.03.003 19616418

[24] Akineden Ö, Annemüller C, Hassan AA, Lämmler C, Zschöck M, Annemu C (2001). Toxin genes and other characteristics of Staphylococcus aureus isolates from milk of cows with Mastitis. Society.

[25] Arciola CR, Baldassarri L, Montanaro L (2001). Presence of icaA and icaD genes and slime production in a collection of staphylococcal strains from catheter-associated infections. J Clin Microbiol.

[26] Gilot P, Lina G, Cochard T, Poutrel B (2002). Analysis of the genetic variability of genes encoding the RNA III-activating components Agr and TRAP in a population of Staphylococcus aureus strains isolated from. J Clin Microbiol.

[27] Jarraud S, Mougel C, Thioulouse J (2002). Relationships between Staphylococcus aureus genetic background, virulence factors, agr groups (alleles), and human disease. Infect Immun.

[28] Clinical and Laboratory Standards Institute: M100-S23 Performance Standards for Antimicrobial Susceptibility Testing; Twenty-Third Informational Supplement. 2013.

[29] Magiorakos A, Srinivasan A (2012). Multidrug-resistant, extensively drug‐resistant and pandrug‐resistant bacteria: an international expert proposal for interim standard definitions for acquired resistance. Eur J Clin Microbiol Infect Dis.

[30] Enright MC, Day NPJ, Davies CE, Peacock SJ, Spratt BG (2000). Multilocus Sequence Typing for characterization of Methicillin-Resistant and Methicillin-Susceptible clones of Staphylococcus aureus. J Clin Microbiol.

[31] Ramakant P, Verma AK, Misra R, Prasad KN, Chand G, Mishra A, Agarwal G, Agarwal A, Mishra SK. Changing microbiological profile of pathogenic bacteria in diabetic foot infections: Time for a rethink on which empirical therapy to choose? Diabetologia. 2011;54:58–64. 10.1007/s00125-010-1893-7 20835702

[32] Shankar EM, Mohan V, Premalatha G, Srinivasan RS, Usha AR (2005). Bacterial etiology of diabetic foot infections in South India. Eur J Intern Med.

[33] Sekhar S, Vyas N, Unnikrishnan M, Rodrigues G, Mukhopadhyay C (2014). Antimicrobial susceptibility pattern in diabetic foot ulcer: A pilot study. Ann Med Health Sci Res.

[34] Tentolouris N, Petrikkos G, Vallianou N, Zachos C, Daikos GL, Tsapogas P, Markou G, Katsilambros N. Prevalence of methicillin-resistant Staphylococcus aureus in infected and uninfected diabetic foot ulcers. Clin Microbiol Infect. 2006;12:186–9. 10.1111/j.1469-0691.2005.01279.x 16441460

[35] Sotto A, Lina G, Richard J (2008). Virulence potential of Staphylococcus aureus strains isolated from diabetic foot ulcers. Diabetes Care.

[36] Gadepalli R, Dhawan B, Sreenivas V, Kapil A, Ammini AC, Chaudhry R (2006). A clinico-microbiological study of diabetic foot ulcers in an Indian tertiary care hospital. Diabetes Care.

[37] Swarna SR, Madhavan R, Gomathi S, Thamaraiselvi S (2012). A study of Biofilm on Diabetic Foot Ulcer. Int J Res Pharm Biomed Sci.

[38] Sader HS, Pritsche TR, Kaniga K, Ge Y, Jones RN (2005). Antimicrobial activity and spectrum of PPI-0903 m (T-91825), a novel cephalosporin, tested against a worldwide collection of clinical strains. Antimicrob Agents Chemother.

[39] Goldstein EJC, Citron DM, Merriam CV, Tyrrell KL (2013). Comparative in vitro activity of ceftaroline, ceftaroline-avibactam, and other antimicrobial agents against aerobic and anaerobic bacteria cultured from infected diabetic foot wounds. Diagn Microbiol Infect Dis.

[40] Lipsky BA (2015). Ceftaroline fosamil for treatment of diabetic foot infections: the CAPTURE study experience. Diabetes Metab Res Rev.

[41] Zhu W, Tenover FC, Limor J, Lonsway D, Prince D, Dunne WM, Patel JB (2006). Use of pyrosequencing to identify point mutations in domain V of 23S rRNA genes of linezolid-resistant Staphylococcus aureus and Staphylococcus epidermidis. Eur J Clin Microbiol Infect Dis.

[42] Malik A, Mohammad Z, Ahmad J (2013). The diabetic foot infections: biofilms and antimicrobial resistance. Diabetes Metab Syndr.

[43] Citron DM, Goldstein EJC, Merriam CV, Lipsky BA, Abramson MA (2007). Bacteriology of moderate-to-severe diabetic foot infections and in vitro activity of antimicrobial agents. J Clin Microbiol.

[44] Melo-Cristino J, Resina C, Manuel V, Lito L, Ramirez M (2013). First case of infection with vancomycin-resistant Staphylococcus aureus in Europe. Lancet.

[45] Saravolatz LD, Pawlak J, Johnson LB (2012). In vitro susceptibilities and molecular analysis of vancomycin-intermediate and vancomycin-resistant Staphylococcus aureus isolates. Clin Infect Dis.

[46] Campion JJ, McNamara PJ, Evans ME (2004). Evolution of ciprofloxacin-resistant Staphylococcus aureus in in vitro pharmacokinetic environments. Antimicrob Agents Chemother.

[47] Martineau F, Picard FJ, Lansac N, Ménard C, Roy PH, Ouellette M, Bergeron MG (2000). Correlation between the resistance genotype determined by multiplex PCR assays and the antibiotic susceptibility patterns of Staphylococcus aureus and Staphylococcus epidermidis. Antimicrob Agents Chemother.

[48] Zubair M, Malik A, Ahmad J, Rizvi M (2011). A study of biofilm production by gram negative organisms isolated from diabetic foot ulcer patients. Biol Med.

[49] Shakeri F, Shojai A, Golalipour M, Alang SR, Vaez H, Ghaemi EA (2010). Spa diversity among MRSA and MSSA strains of Staphylococcus aureus in north of Iran. Int J Microbiol.

[50] Mottola C, Mendes JJ, Cristino JM, Cavaco-Silva P, Tavares L, Oliveira M (2015). Polymicrobial biofilms by diabetic foot clinical isolates. Folia Microbiol (Praha).

[51] Hartford O, O’Brien L, Schofield K, Wells J, Foster T (2001). The Fbe (SdrG) protein of Staphylococcus epidermidis adherance to fibrinogen. Microbiology.

[52] Vandenesch F, Naimi T, Enright MC, Lina G, Nimmo GR, Heffernan H, Liassine N, Bes M, Greenland T, Reverdy ME, Etienne J. Community-acquired methicillin-resistant Staphylococcus aureus carrying panton-valentine leukocidin genes: Worldwide emergence. Emerg Infect Dis. 2003;9:978–84. 10.3201/eid0908.030089 PMC3020611 12967497

[53] Nazareth R, Gonçalves-Pereira J, Tavares A, Miragaia M, de Lencastre H, Silvestre J, Freitas P, Gonçalves E, Martins F, Mendes V, Tapadinhas C, Póvoa P. Infeção por staphylococcus aureus meticilina-resistente da comunidade em Portugal. Rev Port Pneumol. 2012;18:34–8. 10.1016/j.rppneu.2011.05.007 21802892

[54] Francois P, Koessler T, Huyghe A, Harbarth S, Bento M, Lew D, Pittet D, Schrenzel J: Rapid Staphylococcus aureus agr type determination by a novel Multiplex Real-Time Quantitative PCR assay. J Clin Microbiol 2006. 10.1128/JCM.44.5.1892-1895.2006 PMC1479209 16672433

[55] Machuca MA, Sosa LM, González CI (2013). Molecular typing and virulence characteristic of methicillin-resistant Staphylococcus aureus isolates from pediatric patients in Bucaramanga, Colombia. PLoS One.

[56] Traber KE, Lee E, Benson S, Corrigan R, Cantera M, Shopsin B, Novick RP. agr function in clinical Staphylococcus aureus isolates. Microbiology. 2008;154(Pt 8):2265–74. 10.1099/mic.0.2007/011874-0 PMC4904715 18667559

[57] Tenover FC, Arbeit RD, Goering RV, Mickelsen PA, Murray BE, Persing DH, Swaminathan B. Interpreting chromosomal DNA restriction patterns produced by pulsed-field gel electrophoresis : criteria for bacterial strain typing. J Clin Microbiol. 1995;33:2233–9. 10.1128/jcm.33.9.2233-2239.1995 PMC228385 7494007

[58] Monecke S, Coombs G, Shore AC, Coleman DC, Akpaka P, Borg M, Chow H, Ip M, Jatzwauk L, Jonas D, Kadlec K, Kearns A, Laurent F, O’Brien FG, Pearson J, Ruppelt A, Schwarz S, Scicluna E, Slickers P, Tan HL, Weber S, Ehricht R. A field guide to pandemic, epidemic and sporadic clones of methicillin-resistant Staphylococcus aureus. PLoS One. 2011;6:e17936. 10.1371/journal.pone.0017936 PMC3071808 21494333

[59] Aires-de-Sousa M, Correia B, De Lencastre H, Alves V, Branca F, Cabral L, Clemente J, Daniel I, Faustino A, Ferreira E, Lameiras C, Lopes J, Marques J, Peres I, Ribeiro G, Sancho L, Santos O, Santos P, Vaz MT, Videira Z. Changing patterns in frequency of recovery of five methicillin-resistant Staphylococcus aureus clones in Portuguese hospitals: Surveillance over a 16-year period. J Clin Microbiol. 2008;46:2912–7. 10.1128/JCM.00692-08 PMC2546730 18614664

[60] Monecke S, Ehricht R (2005). Rapid genotyping of methicillin-resistant Staphylococcus aureus (MRSA) isolates using miniaturised oligonucleotide arrays. Clin Microbiol Infect.

[61] Tavares A, Miragaia M, Rolo J, Coelho C, De Lencastre H (2013). High prevalence of hospital-associated methicillin-resistant Staphylococcus aureus in the community in Portugal: Evidence for the blurring of community-hospital boundaries. Eur J Clin Microbiol Infect Dis.

[62] Espadinha D, Faria NA, Miragaia M, Lito LM, Melo-Cristino J, de Lencastre H, Network MS. Extensive dissemination of Methicillin-Resistant Staphylococcus aureus (MRSA) between the hospital and the community in a country with a high prevalence of nosocomial MRSA. PLoS One. 2013;8:1–8. 10.1371/journal.pone.0059960 PMC3617237 23593155

[63] Sakwinska O, Kuhn G, Balmelli C, Francioli P, Giddey M, Perreten V, Riesen A, Zysset F, Blanc DS, Moreillon P. Genetic diversity and ecological success of staphylococcus aureus strains colonizing humans. Appl Environ Microbiol. 2009;75:175–83. 10.1128/AEM.01860-08 PMC2612194 18978084

[64] Fan J, Shu M, Zhang G, Zhou W, Jiang Y, Zhu Y, Chen G, Peacock SJ, Wan C, Pan W, Feil EJ. Biogeography and virulence of Staphylococcus aureus. PLoS One. 2009;4:e6216. 10.1371/journal.pone.0006216 PMC2705676 19593449

